# Nuclear envelope budding and its cellular functions

**DOI:** 10.1080/19491034.2023.2178184

**Published:** 2023-02-22

**Authors:** Katharina S. Keuenhof, Verena Kohler, Filomena Broeskamp, Dimitra Panagaki, Sean D. Speese, Sabrina Büttner, Johanna L. Höög

**Affiliations:** a Department for Chemistry and Molecular biology, University of Gothenburg, Sweden; b Institute of Molecular Biosciences, University of Graz, Austria; c Department of Molecular Biosciences, The Wenner-Gren Institute, Stockholm University, Sweden; d Knight Cancer Early Detection Advanced Research Center, Oregon Health and Science University, 2720 S Moody Ave, Portland, OR, 97201, USA

**Keywords:** Nuclear import, nuclear export, nuclear envelope budding

## Abstract

The nuclear pore complex (NPC) has long been assumed to be the sole route across the nuclear envelope, and under normal homeostatic conditions it is indeed the main mechanism of nucleo-cytoplasmic transport. However, it has also been known that e.g. herpesviruses cross the nuclear envelope utilizing a pathway entitled nuclear egress or envelopment/de-envelopment. Despite this, a thread of observations suggests that mechanisms similar to viral egress may be transiently used also in healthy cells. It has since been proposed that mechanisms like nuclear envelope budding (NEB) can facilitate the transport of RNA granules, aggregated proteins, inner nuclear membrane proteins, and mis-assembled NPCs. Herein, we will summarize the known roles of NEB as a physiological and intrinsic cellular feature and highlight the many unanswered questions surrounding these intriguing nuclear events.

## Introduction

Before the appearance of eukaryotic cells, membranes were present only in the form of the plasma membrane that defined the cell’s borders, whereas its internal environment lacked compartmentalization. About 1.5 billion years ago, one of the most defining evolutionary steps occurred, leading to the formation of an endomembrane system and a sophisticated cellular compartmentalization. Eukaryotic cells benefit from this compartmentalization that, among other things, enables them to maintain incompatible biochemical reactions simultaneously through division into distinct membrane-delineated environments.

The nucleus is one of the largest organelles of the cell [1-5], carrying out many different cellular functions. It contains nuclear DNA and is the site for DNA replication, transcription, and post-transcriptional modification of mRNA [6]. Until recently, nuclear pore complexes (NPCs) were considered the only route in and out of the nucleus for endogenous proteins and RNA. This mode of nuclear export is highly regulated, intensely studied, and indeed a major player in nucleo-cytoplasmic transport [7]. Another pathway, known as nuclear egress or envelopment/de-envelopment has long been accepted as a route of herpesviral escape from the nucleus [8,9] but was proposed to be specific to herpesviruses and not an endogenous cellular transport mechanism. However, over the past 70 years of research, there have been hints of similar mechanisms being utilized in non-infected cells [10–26], but these studies were mainly at the observational level and lacked mechanistic insights. More recently, nuclear envelope budding (NEB) was proposed to be an endogenous mechanism of nuclear transport and shown to be present in all organisms investigated under normal growth conditions [27], suggesting that NEB-like mechanisms were likely co-opted by herpesviruses for nuclear egress. Recent studies have suggested that NEB can serve to transport ribonucleoproteins (RNPs) [28,29], protein aggregates [27] and inner nuclear membrane components [30] (also reviewed in [31]). Discovery and characterization of NEB has until now occurred via ultrastructural analysis. The unavailability of a NEB-specific fluorescent marker has prevented the unequivocal visualization of transport across the nuclear envelope in a time-lapse movie. This lack of live imaging is currently the biggest obstacle to the progression of the investigation of NEB events. In this review, we will summarize the state of knowledge around these mostly overlooked but potential nucleo-cytoplasmic transport pathways and discuss the known and potential implications of them in aging, cancer, and neurodegenerative diseases.

## A neglected child with many names?

It is important to note that the terminology for NEB-like nuclear envelope dynamics observed over the years is quite diverse, which can make it hard when performing literature searches to uncover previous observations. Moreover, the determination of common nuclear envelope dynamics for pathways that seem to have distinct molecular functions is complex. While not an exhaustive list, dynamics of the nuclear envelope similar to NEB have been referred to as nuclear blebbing [11], nuclear or nucleolar – where presumably the nucleolus itself also protrudes into the cytosol – extrusion [10,32], nucleo-cytoplasmic relations [21,33], nucleo-cytoplasmic interactions [18,20], nuclear envelope (NE) budding [28,34], outpocketing of the nuclear envelope [21], and nuclear envelope herniations [35]. As it is still the early days of mechanistically dissecting these pathways, it should be cautioned that it is not clear how all these various nuclear envelope dynamics interrelate and whether they represent pathways that have overlapping morphological features but distinct molecular functions. Definitive experiments to unequivocally prove that all NEB and NEB-like pathways represent a mechanism to transfer material over the nuclear envelope have not been conducted. That said, the existing evidence strongly suggests that multiple alternative mechanisms of nucleo-cytoplasmic transfer exist. Indeed, it is almost certain that NPC-mediated transport accounts for the vast majority of nuclear transport in the cell [36–38] (and reviewed in [39]) and that these alternative modes of transport are more utilized during distinct development stages and under pathological conditions. However, evidence from the herpesviral egress field alone is enough to make a strong argument that alternative endogenous modes of nuclear export exist. Herein, we define NEB events as a visible deformation and separation of nuclear membranes, with cargo usually surrounded by membranes, contained within the perinuclear space. As the field moves forward, we can hopefully start to parse out distinct molecular pathways and assign clear nomenclature to the various pathways that are emerging.

## The nuclear pore complex: the only route of transport across the nuclear envelope?

A vital component of the nucleus is the nuclear envelope, which separates its contents from the rest of the cell. Both the inner (INM) and outer (ONM) nuclear membranes consist of a phospholipid bilayer that contains a plethora of membrane proteins [40,41], making it a dynamic and crowded environment. The space between the INM and ONM is referred to as the perinuclear space. This nuclear enclosure provides isolation of the genome from many sources of damage. However, due to these separating membranes, the existence of mechanisms that ensure an immaculate flux of metabolites, RNA, and proteins is required.

NPCs have long been assumed to be the sole means of transport of molecules across the nuclear envelope [36,38]. Central to all eukaryotic life, NPCs mediate bidirectional communication between the nucleoplasm and the cytoplasm. They maintain the integrity of the nuclear compartment by preventing macromolecules from diffusing freely in and out of the nucleus [42]. When macromolecules are translocated through the NPCs, it is a very rapid process, occurring at a rate of about 1000 translocations per second [43].

NPCs are among the largest protein complexes found in cells, with an estimated mass of 60 MDa in yeast and 120 MDa in vertebrates [44–47]. They consist of multiple copies of over 30 unique proteins, termed nucleoporins (Nups), with the exact number of Nups being species-dependent [48–50]. With their eightfold symmetry, NPCs consist of a core ring embedded in the nuclear envelope, two outer rings, one cytoplasmic ring, and eight attached nuclear filaments [51]. The nuclear filaments are joined in a distal ring assembling as the nuclear basket [44,51,52].

Each NPC resides at and stabilizes an ~80 nm-wide pore with an inner diameter of ~40 nm that is generated by fusion of the INM and ONM [44,53]. Due to the channel size, NPCs can transport small molecules in their natively folded state, which means that these molecules remain functional [44]. While small molecules such as metabolites and ions pass freely through the NPC, the diffusion of larger molecules is restricted and governed by their size and surface properties [44]. This size limit is not a hard cutoff, rather the energetic barrier for passive diffusion increases rapidly for molecules larger than 40 kDa, but molecules as large as 60–100 kDa can pass through the NPC [54–56]. However, large macromolecular assemblies that greatly exceed the size limit of the 40 nm pore, such as whole virions (100–300 nm), RNA granules (average of 200 nm), or large protein aggregates (variable but up to several hundred nm) have been proposed to be transported across the nuclear envelope and thus the idea of an alternative nuclear export pathway needs to be considered.

## The slow discovery of nuclear envelope budding pathways

**Figure 1.**
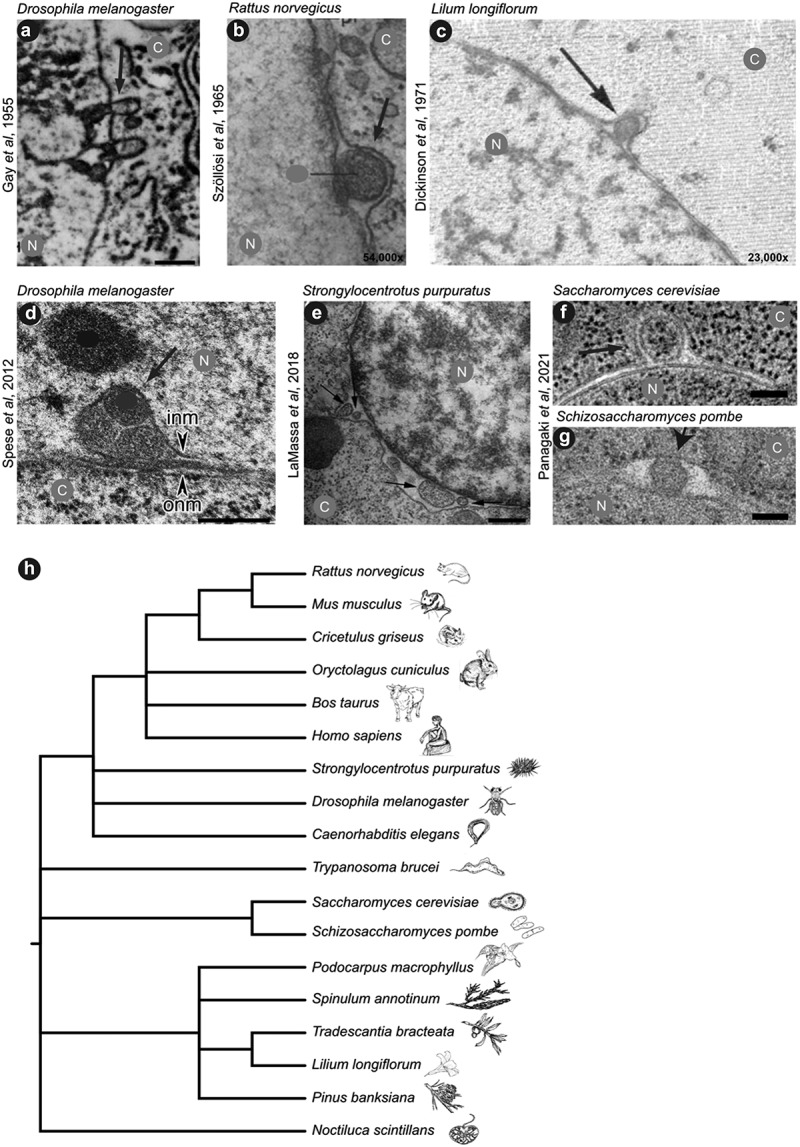
A selection of NEB events from different species found in literature over the past 70 years. a) Electron micrograph of a fruit fly cell [21]. b) Rat oocytes 54,000x magnification [10]. c) Microspores from an Easter lily. 23,000x [20]. d) *Drosophila* larval muscle nuclei with arrowheads pointing to inner nuclear membrane (INM) and outer nuclear membrane (ONM) [28]. e) Sea urchin gastrula [13]. f) Slice from an electron tomogram of a NEB event in budding yeast. g) Thin section of a fission yeast cell [27]. Scale bars in panels a, d, and e are 500 nm and in panels f and g are 100 nm, scale bars were unavailable in the original publications of panels b and c; N: nucleus, C: cytosol. Arrows are pointing to nuclear budding events, other labels from the original publications were hidden (grey and black dots) to ease comprehension of the adapted figure. (h) Phylogenetic tree of species in which NEB and NEB-like mechanisms has been observed, adapted from [27].

Numerous electron microscopy studies, some dating back to the early 1950s, have described NEB events under various names [10–12,14–21,23] (Figure 1a-g). NEB-like events have now been seen in about 20 different species within all kingdoms of eukaryotic life. Although most events are protrusions of the ONM into the cytoplasm (Figure 1a-c, e and f), some variations of this structure include a protrusion of the INM into the nucleoplasm (Figure 1d) [23]. The shape of these NEB events can differ, ranging from spherical (Figure 1b and d) to elongated (Figure 1a, c and e). Occasionally, the perinuclear content is also exactly between the INM and ONM, giving it the appearance of an eye (Figure 1g). NEB events also vary in electron density and appearance. The cargo contained in the perinuclear space is often surrounded by a membrane and may be of similar texture to the nucleoplasm. Nevertheless, NEB events occasionally contain material that resembles cytosol, including ribosomes [27], suggesting bidirectionality of this transport route across the nuclear envelope.

One of the first studies that described NEB events was performed in third instar larvae of the fruit fly (*Drosophila melanogaster*) in 1955 [21]. In disbelief of their initial observations, several attempts were made to improve the sample preparation protocol, without successfully eliminating the presence of such nuclear blebs. Instead, it was hypothesized that these ‘outpocketings’ were probably a manifestation of normal cellular function with two main theories around their existence. First, the authors suggested the cargo of these events to be strongly associated with the transport of some sort of genetic material from the nucleus to the cytoplasm. Second, they proposed that the protruding membrane could serve as a new membrane component of the endoplasmic reticulum (ER). Common to both theories was the assumption that the shape and composition of these blebs would rapidly change upon release into the cytoplasm.

Several other studies then have made similar observations as well as theories behind these events [13,25,28,29,57–60]. These studies were also performed in several different organisms, implying an evolutionarily conserved nature of this process (Figure 1h). The first publication that reached beyond the observational stage and described the nature of the cargo and the physiological occurrence of these events, was performed in *D. melanogaster* by Speese *et al*. in 2012 [28]. In their study, the authors proposed that the transported cargo contained large ribonucleoprotein (RNP) granules, which, after exiting the nucleus through NEB, translocated to specific sites where synapse formation normally takes place. This translocation step has yet to be directly demonstrated. Importantly, later findings from the Budnik lab provided first insights into a mechanism that connects NEB with muscular dystrophies and related disorders, which we will discuss further in the disease-related chapter below [57,58]. To date, several reports of NEB events occurring during normal cellular physiological pathways have been made [27,28,59]. In support of these results, we recently showed that NEB events occur during normal growth conditions, by examining five different organisms across the eukaryotic domain: the protist *Trypanosoma brucei*, the two yeast species *Saccharomyces cerevisiae* and *Schizosaccharomyces pombe*, the nematode *Caenorhabditis elegans,* and a human mast cell line (HMC-1) [27]. This strengthens the conclusion that budding of the nuclear envelope represents a normal cellular function conserved across species barriers, but also invokes the possibility that morphologically similar events in the nuclear membrane could be transporting different types of cargo and thus could represent distinct molecular pathways that just happen to share overlapping nuclear envelope dynamics.

To date, these observations have been completely dependent on the use of electron microscopy as a means of capturing snapshots of this transport process. More recently, different molecular markers have also been used to study NEB in light microscopy. These markers include nuclear localization signals fused to a fluorophore, other proteins found at the nuclear surface, or viral nuclear egress proteins [28,30,34,61–64]. However, only a few studies have thoroughly correlated their light microscopy markers, for example, nuclear lamins in *D. melanogaster* [28], Atg39 in *S. cerevisiae* [30], or viral proteins [63,65,66] with NEB events within the perinuclear space using electron microscopy, which is needed to definitively identify NEB events. Optimizing markers to study NEB and the use of super-resolution light microscopy will be essential to progress and provide a mechanistic understanding of this exciting research field.

## Herpesviral egress from the nucleus

**Figure 2.**
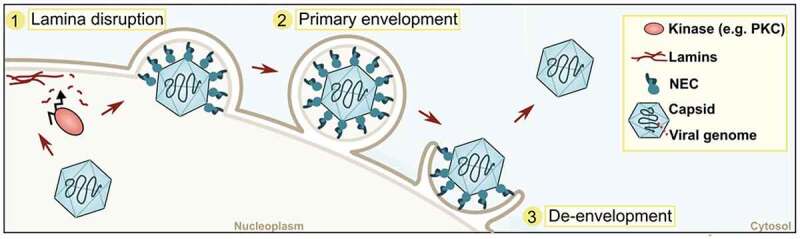
Scheme of herpesviral egress. Three main steps during the envelopment/de-envelopment process of Herpesviridae are depicted. NEC: nuclear egress complex, PKC: protein kinase C.

Most DNA viruses and a set of RNA viruses exploit the host cell’s nuclear DNA replication and transcription machinery for their own transcription and genome replication (reviewed in [67–71]). To make use of these cellular machineries, the nuclear envelope needs to be crossed twice, once for import of the viral DNA and once for exit of newly assembled virions [68]. Here, we focus on how newly formed herpesviruses leave the nucleus after viral replication, as this occurs in a manner similar to NEB (Figure 2) [72–74].

The herpesvirus family consists of numerous different viruses infecting vertebrates and invertebrates, with the common morphology of an icosahedral capsid enclosing linear double-stranded DNA that is surrounded by a membrane [75]. After DNA replication, transcription and translation, new capsids containing the viral genome are assembled in the nucleus and then employ a specialized mechanism for nuclear and cellular egress. Nuclear egress requires specific viral proteins (extensively reviewed in [76–78]). In short, two conserved viral proteins (e.g., UL31 and UL34 for HSV-1) form a heterodimer that together with host-specific proteins [65] builds a nuclear egress complex (NEC) ([79–81] and reviewed in [82]). The NEC controls capsid envelopment at the INM. To that end, the nuclear lamina, a dense filamentous protein network underneath the INM that provides shape and mechanical stability (reviewed in [83]), requires (partial) disruption of the viral capsid to gain access to the NEC and to bud at the INM (Figure 2). The nuclear lamina is disrupted [84] by recruitment of host Protein Kinase C (PKC)/viral kinases (e.g., US3 for HSV-1) which locally phosphorylate components of the nuclear lamina, resulting in alterations that enable the capsid to access the INM [85–88]. This mimics the host cell’s kinase activity that occurs during nuclear envelope breakdown in the cell cycle and thus promotes nuclear egress of viral capsids [89]. The NEC not only governs recruitment of capsid proteins to the INM but also facilitates membrane curvature during viral budding [66,90–92]. Host ESCRT-III (endosomal sorting complex required transport III) machinery is proposed to be required for vesicular scission, ensuring the budding off of membranes containing capsids into the perinuclear space (primary envelopment) [93].

These perinuclear enveloped viruses then fuse with the ONM to release naked viral capsids into the cytosol – a process called de-envelopment (Figure 2). It is assumed that, among other viral and host factors, viral kinases (US5 for HSV-1) play an important role during this process [65,94,95]. However, the fusogen that drives fusion of the INM and ONM has remained elusive. This mechanism of crossing the nuclear envelope via envelopment/de-envelopment steps has long been assumed to be unique to herpesvirus trafficking. However, as viruses are known to highjack normal cellular functions to their own advantage, it is not surprising that cells are using this route to transport their own cargo and that NEB represents a physiological and intrinsic feature of all cells.

## The nature of NEB cargo

A key question, of course, is: what is possibly being transported across the nuclear envelope in these NEB events in a healthy growing cell? In the following sections, we will elaborate on the different cargoes so far identified that are all products the nucleus needs to dispose of. This list will likely grow as the field progresses.

## Transport of RNA granules

**Figure 3.**
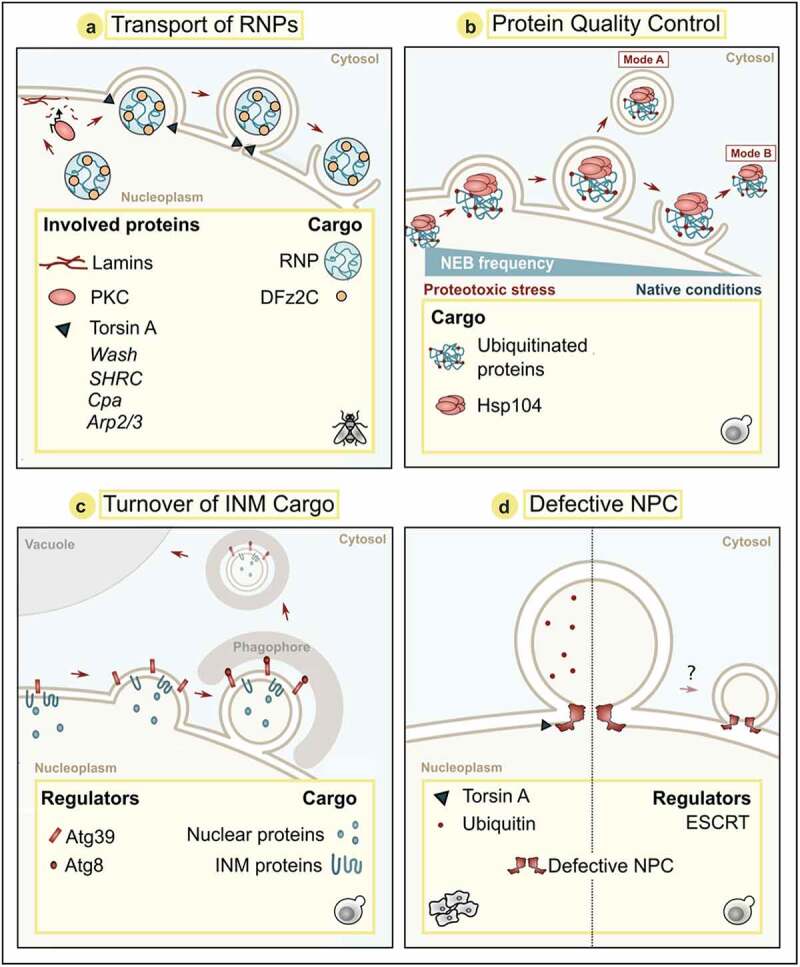
The nature of NEB cargo. Schemes of four modes of nuclear envelope budding events with known cargos and regulators. Panel b shows two modes: (Mode a) Double-membrane vesicle carrying cargo after budding event; (Mode b) Cargo released without membrane enclosure. Organisms, where these modes of actions were described are panel a: *Drosophila melanogaster*; panels b, c, d (right): *Saccharomyces cerevisiae*; panel d (left): Mammals. NPC: Nuclear pore complex, PKC: Protein Kinase C.

Due to ‘the intimate association between chromosomal material and membrane outpocketings revealed in these electron micrographs’ [21] (Figure 1a), Gay first suggested that the NEB events might be transporting a nucleic acid from the nucleus to the cytoplasm. A little over 30 years later, Hochstrasser and Sedat [12] made similar observations and suggested the cargo might be aggregates of RNPs [12]. The first publication positively identifying the cargo being transported through this pathway was then published about 60 years after the first observation [28] (Figure 1d). When studying synaptic signaling in *D. melanogaster*, Speese *et al.* observed that the C-terminus of the protein Frizzled-2 (DFz2C) forms foci at the periphery of the nucleus (Figure 3a). These foci are surrounded by A- and B-type lamins (LamC and LamDm0, respectively). Just like in the process of herpesviral egress via NEB, PKC is also required for the formation of these NEB events, as it causes the partial dissolution of the filamentous lamin network. This is a prerequisite to move large cargo through the otherwise small fenestrations in the nuclear lamina.

Ultrastructural analyses revealed that these foci correspond to NEB events where the INM invaginates into the nucleoplasm, thus creating an enlarged perinuclear space that the RNA granules can bud into. It has later been proposed that the AAA+ ATPase Torsin A found in the perinuclear space is required for membrane scission [60]. The cargo within these invaginations consists of RNA granules of around 200 nm in diameter that are bound by a membrane [28]. RNA granules contain transcripts encoding for various scaffolding proteins required for assembly of the postsynaptic domain of the neuromuscular junction synapse [28,96]. It is still unclear whether these granules contain multiple copies of single or several species of RNA, but it is postulated that RNA granules allow for localized translation of their mRNAs, e.g., at postsynaptic sites. The RNA granules contained within the NEB events were hypothesized to be delivered to other parts of the cell via fusion with the ONM, as shown by their occurrence in the cytoplasm; however, it cannot be ruled out that the transcripts contained are bound for degradation.

Using Fz2C foci as a marker for nuclear envelope buds containing RNA granules, the *Drosophila* washout (WASH) protein, its regulatory complex (SHRC), capping protein Cpa and the Arp2/3 protein complex were identified as players of the molecular machinery underlying NEB in salivary glands of *D. melanogaster* by Verboon *et al*. [34] (Figure 3a). WASH belongs to the Wiskott-Aldrich Syndrome Protein (WASP) family [97] that all have well-established roles in cytoskeleton reorganization. While most members of this protein family function in the cytoplasm, WASH has also been shown to be present in the nucleus, where it interacts with B-type lamins [34]. Genetic modifications that interfere with the function of WASH, SHRC subunits, or Arp2/3 decrease NEB frequency, which was associated with disrupted integrity of the neuromuscular junction and decreased mitochondrial activity [34]. Verboon *et al*. suggest that WASH and SHRC act early in the NEB pathway, upstream of Arp2/3 and Torsin A. Interestingly, a point mutation in WASH that precludes its interaction with Arp2/3 (but does not affect other WASH functions) results in reduced NEB frequency. As Arp2/3 is critical for actin nucleation activity of WASH, the authors propose that WASH might contribute to NEB via modulation of the cortical actin filaments [34]. How exactly these regulatory complexes may be functioning to drive membrane or cortical cytoskeleton remodeling during NEB is yet to be understood. It will also be interesting to see if the function of these complexes in NEB and other nuclear envelope remodeling events is conserved in other species.

## NEB to transport protein aggregates

Rose *et al.* [98] proposed already in 2012 that NEB-like mechanisms may also be used as a pathway to clear the nucleus of aggregated proteins. Misfolded proteins are marked with ubiquitin as a flag for the proteasome to degrade it, as reviewed in [99]. When the removal of misfolded proteins via the ubiquitin-proteasome system fails, terminally damaged and aggregated proteins can be sequestered into insoluble inclusions, reviewed in [100]. This is called spatial protein quality control and represents an intrinsic feature of the cellular stress response, preventing proteotoxicity of accumulating misfolded proteins [101–103]. There are several locations where cells sequester protein aggregates, e.g., the intranuclear and/or juxtanuclear quality control compartments (INQ/JUNQ), the vacuole-associated insoluble protein deposit (IPOD), and the cytoplasmic CytoQ in yeast or the aggresome in mammalian cells [102–104]. Thus, a damaged and misfolded protein within the nucleoplasm can either be removed via the proteasome or, if this fails, be terminally sequestered into INQ (reviewed in [105]). New evidence suggests that upon proteotoxic stress or proteasome inactivation, aggregated proteins can also be transported across the nuclear membrane via NEB [27]. Our research groups investigated the presence and frequency of NEB events under different cellular stress conditions in the budding yeast *S.cerevisiae* (Figure 3b). Interestingly, different stress conditions, such as heat shock, arsenite, hydrogen peroxide, proteasomal inhibition, and treatment with the proline analog azetidine-2-carboxylic acid, which promotes protein misfolding, significantly increase the frequency of NEB events. These observations, in combination with the presence of ubiquitin and the protein disaggregase Hsp104 inside the forming buds, established a connection between NEB and the protein quality control system.

## NEB to sequester INM cargo

Recent evidence suggests that NEB-like mechanisms also contribute to the turnover of damaged INM components via a selective mode of autophagy termed nucleophagy [30, 107] (reviewed in [106]). In yeast, autophagy can be induced when cells are starved of nitrogen or treated with the drug rapamycin. Both treatments resulted in nuclear envelope protrusions [107] and NEB [30] followed by vacuolar removal of INM and nuclear cargo (Figure 3c). The autophagy receptor Atg39 is a transmembrane protein that spans the ONM [108] and connects to the luminal leaflet of the INM via membrane-binding amphipathic helices [30,107]. Atg39 contributes to membrane bending and capturing of INM cargo and nuclear material into NEBs. Interestingly, NPCs seem to be excluded from the regions of NEB in this pathway, and thus this likely represents a distinct selective quality control pathway [109]. Ultrastructural investigation suggests that this process might occur with two mechanistic variations [30,107]. Upon accumulation of Atg39 at the ONM, a simultaneous protrusion of the INM and ONM outwards has been observed, releasing a nuclear envelope-vesicle with a double bilayer in a single scission event [107]. Alternatively, Atg39-captured INM cargo can be sequestered into an INM vesicle that is first released into the perinuclear space, requiring a first membrane scission event at the INM. This is followed by a protrusion outwards and a second scission event at the ONM that also releases a double bilayer vesicle for subsequent autophagic removal [30]. Whether these nuclear envelope remodeling events observed during nucleophagy correspond to those formed upon stress and proteasomal inhibition [27] remains to be shown, but such double bilayer vesicles have not been observed in the cytoplasm of stressed cells. On the other hand, as Atg39 is expressed specifically upon cell stress [110] and overexpression of Atg39 is sufficient to induce INM cargo sequestration and NEB formation [30], a connection seems likely.

## Nuclear envelope herniations in response to defective NPC assembly

Transportation through the NPC is a highly efficient and regulated mechanism for nucleo-cytoplasmic communication, and so it is not surprising that malfunction of this process is fatal for the cell. *S. cerevisiae* for example has dedicated protein quality control mechanisms, one of them being the strategy of keeping dysfunctional NPCs in the mother cells via asymmetric segregation of cell material during budding [35,109]. This ensures that daughter cells begin their lives with only fully functional, recently synthesized proteins. This is achieved by only passing short-lived basket Nups but not long-lived core Nups to the offspring [111]. The protein Nsp1 represents a crucial node in this NPC quality control pathway as it senses their functionality and regulates their transmission into progeny, most likely via a bud neck diffusion barrier [112–115]. Nuclear envelope herniations very similar to NEB events have been observed in response to compromised NPCs. These herniations form upon deletion or mutation of genes encoding Nups or NPC-associated factors [109,116–122], as comprehensively summarized and discussed in a recent review [31].

Assembly of the NPC in the NE occurs stepwise. Starting at the INM, the different subcomplexes are sequentially recruited, the INM is protruding into the perinuclear space, the INM and ONM fuse and the cytosolic filaments are added (reviewed in [123]). Malfunctioning of this assembly process drives the formation of hernia in the INM. These omega-shaped structures cause the ONM to protrude toward the cytosol while often remaining associated with the INM. Here, partially assembled NPCs are found (reviewed in [31]), as recently confirmed by cryo-ET followed by sub-tomogram averaging in yeast, revealing its structure (Figure 3d) [124].

It remains to be explored whether these nuclear envelope herniations that occur in response to defects in NPC assembly are formed by similar mechanisms and are functionally connected to the NEB events containing other cargoes. Interestingly, the nuclear egress of herpesvirus, the proposed nucleo-cytoplasmic shuttling of RNA granules via NEB in *Drosophila* muscle and salivary gland, as well as NPC-containing herniations share Torsin A as common molecular determinant [60,125,126]. In human cell lines, the lack of Torsin A or its cofactors has been shown to induce herniations that contain NPC assembly intermediates at their neck [127–129]. Those herniations carry ubiquitin, just as the stress-induced NEB events in yeast [27]. How exactly the loss of Torsin A disrupts NPC assembly, thereby leading to herniation formation, is not yet known.

Nuclear envelope herniations have also been linked to the quality control of already fully assembled but damaged NPCs (reviewed in [31]). Formation of herniations in this context seems to involve membrane remodeling by the ESCRT machinery and INM proteins of the Lap2-emerin-MAN1 (LEM) family [109,122,130,131]. In a yeast mutant lacking Nup116, phosphatidic acid-rich membrane stretches and an interaction with LEM proteins recruits ESCRT components to the nuclear envelope herniations [122,132], but the precise role of these proteins at the herniations remains to be explored.

The fate of the nuclear envelope herniations containing defective NPCs remains unclear. One could imagine it as either a mere storage site for a damaged NPC, or a first step in a degradation pathway. Two lines of evidence suggest that this might be a matter of storage. First, the degradation of NPCs upon starvation-induced autophagy was reduced in the herniation-forming mutant *nup116Δ* [124]. Second, NPCs have even been shown to be excluded from Atg39-associated NEB events that facilitate the autophagic turnover of INM cargo [30]. So, what about the nuclear herniations being a first step in a degradation pathway? The partial NPC intermediates within membrane-concealed herniations are not accessible to the autophagy machinery as they are not exposed to the cytoplasm [124]. Such a degradation mechanism would therefore require further steps for example, releasing the herniation as a vesicle between the INM and ONM, a vesicle that is later released into the cytoplasm. Such vesicles trapped between the INM and ONM have occasionally been seen in NPC assembly-defective yeast cells lacking the ESCRT component Vps4 [109]. However, to determine if these vesicles are the subsequent steps of an NPC degradation pathway, one would ideally see the partially assembled NPC or localize its proteins to the same structure. Therefore, future research will have to settle what the function and fate of these nuclear herniations are.

## Imbalances in nuclear-cytoplasmic transport in human disease

One of the most unfailing ways to render a cell dysfunctional is to hinder proper NPC function, a strategy executed by caspases during apoptosis and by viral proteins during infection [133–137]. Thus, NPC building blocks require sophisticated surveillance, sequestration, and removal strategies to ensure cell survival [35,109,113–115,138–142]. Many factors lead to NPC malfunctions and eventual degradation, including the misassembly of newly synthesized NPCs, aggregation of Nups, as well as cellular stress and aging-related diseases (neurodegeneration, cardiovascular diseases, and cancer) [143–148]. Thus, relying solely on the NPC-mediated route for a process as important as the nuclear import and export of proteins, RNA, and ribosomes seems rather risky for the cell. The existence of an alternative nucleo-cytoplasmic gateway makes sense.

NPC-linked health problems are broad and have been reviewed extensively [138,146,149–152]. For example, impaired nucleo-cytoplasmic transport is linked to amyotrophic lateral sclerosis [153,154], frontotemporal dementia, and Huntington’s disease [155]. In Huntington’s disease, NPCs were found decorating the disease-linked poly Q aggregates [156]. In Alzheimer’s disease it was recently reported that Nup98 directly interacts with tau, leading to tau aggregation and Nup sequestration into cytoplasmic neurofibrillary tangles, followed by deterioration of NPCs [157]. Overall, impaired function of NPCs has been strongly connected to neuronal aging and neurodegeneration.

In addition, cardiovascular diseases are also linked to NPC malfunction, as first shown by Zhang *et al.* where a mutation in Nup155 led to atrial fibrillation in humans and mice [158]. Patients who have suffered a heart failure have increased levels of several Nups [159], and in ischemic cardiomyocytes Nup35 level is reduced [160]. Over the years, Nups have also been found to build oncogenic fusion proteins with different partners [161]. For example, Nup98 fusions to Hox9, a family of transcription factors commonly mutated in cancer, were shown to induce leukemic transformations [162–164]. Nup88 is elevated in many cancer lines and tumors [165,166] and overexpression causes intestinal tumors in mice, without affecting nucleo-cytoplasmic transport [167].

If NEB indeed represents an alternative route of nucleo-cytoplasmic transport, its frequency likely increases in cells afflicted by the aforementioned diseases with malfunctioning NPC-mediated transport. Although a truly systemic study is yet to be conducted, many indications listed in the next section are pointing that way.

## NEB in aging and disease

The implications of NEB playing a role in physiological cellular functions are just starting to be unveiled. Below we outline several lines of evidence suggesting that the NEB pathway is involved in cellular aging and age-related diseases.

As yeast cells grow old, nuclear envelope herniations that very much resemble NEB events increase in frequency. In fact, these herniations increased 7.5 times in old cells compared to young controls [35]. The authors associated frequent herniations with damaged NPCs, which may likely be the case. However, further studies are needed to clarify whether some of these age-associated herniations may correspond to NEB events whose task is to clear other material from the aging nuclei.

In humans, a group of diseases commonly associated with aging arise from mutations in genes coding for proteins of the nuclear lamina, collectively referred to as laminopathies. In a *D. melanogaster* model for laminopathies based on lamin C mutations (A-type lamin), the NEB pathway has been shown to be affected [59]. The authors propose that the impaired lamina network upon lamin C mutation prevents RNA granules containing mitochondria-related mRNAs from exiting the nucleus through NEB. In consequence, this causes a deterioration of mitochondrial function in muscle tissue [59]. Overall, the compromised integrity of the lamina network in these flies results in disrupted nuclear envelope morphology, mitochondrial defects, and impaired flight behavior. The incorrect cellular localization of RNA granules has also been connected to neurodegeneration [168]. Both insufficient transport of RNA granules to their final destination in the cell periphery and overaccumulation of RNA granules in the nucleus have been observed in neurodegenerative diseases [168–171]. In samples from patients with the neurodegenerative disorder fragile X tremor ataxia syndrome (FXTAS), nuclear RNA granules have been found to co-localize with lamin A and C [172], resembling the structures shown in Speese *et al.* [28]. How NEB contributes to RNA granule transport and assembly, disassembly, or clearance in neurodegenerative diseases still needs to be elucidated.

Torsin A links NEB to another neurological disease. In humans, mutations of the torsin A gene cause DYT1 dystonia – a disease where the synapses between muscle and neuronal cells do not develop properly, leading to uncontrolled muscular twitching. Interestingly, when the torsin A gene is silenced in *D. melanogaster* muscle cells, RNA granule-containing NEB events accumulate in the perinuclear space [60].

The overexpression of a pathogenic torsin A variant in transgenic mice also resulted in movement defects and perinuclear inclusions (or vesicular structures) which contained ubiquitin, lamin A and Torsin A, very similar to NEB events [24–26,173]. Subsequently, it was shown that loss of torsin A in mice also results in accumulation of NEB events in the perinuclear space [24]. Nuclear buds were observed in all brain regions examined but were enriched in a particular neurodevelopmental window [25].

Not only aged cells rely heavily on protein quality control pathways. Due to rapid proliferation and high protein turnover, cancer cells are sensitized to proteotoxic stress. Thus, the heat shock response is often activated in cancer cells, and some chemotherapies target the proteostasis system [174]. Interestingly, the proteasome inhibitor MG132, also known to induce apoptosis in tumor cells [175], significantly increased the frequency of NEB events in yeast [27]. In human breast cancer cells, the receptor SRC-3/AIB1 (amplified in Breast Cancer-1) was extruded by some form of nuclear budding under normal growth conditions [61]. This indicates that the NEB pathway might also be more active in cancer cells, but ultra-structural investigation is needed to confirm that these events have the same morphology as NEB events outlined above. The deletion of WASH inhibits NEB events in *Drosophila* [34] and the human WASH complex is strongly associated with both cancers and neurodegenerative disorders [176–178], but if or how this is in relation to NEB remains to be investigated.

In summary, alterations in NEB and NEB-like mechanisms are associated with several human diseases, many of which are connected to aging. A better understanding of NEB and NEB-like mechanisms and their respective roles in nucleo-cytoplasmic transport is relevant for the development of innovative therapies.

## Discussion

Evolution often equips cells with redundant systems for important processes to ensure their survival, as is the case for DNA repair and cellular metabolism [179,180]. It is therefore not surprising that nucleo-cytoplasmic transport, an essential function of eukaryotes, would have more than one route. We argue that NEB and NEB-like pathways are hidden in plain sight. They are frequently compared to viral egress, which has long been assumed to be a virus-specific trafficking route, even though viruses usually highjack already existing cellular functions for their replication and infection of other cells, e.g., as the vaccinia virus does [181,182]. We hypothesize that the collective NEB pathways represent a heavily understudied means of endogenous nucleo-cytoplasmic transport and conclude that many questions remain unanswered (Text box 1).

It is without doubt that to help answer at least some of the questions, live cell imaging techniques are required. This will be a difficult task due to the level of spatial and temporal resolution needed. Super-resolution light microscopy techniques for example, using a single-molecule approach, can provide answers if the temporal resolution is high enough and appropriate markers for cargo and membranes can be used. Correlation with an ultrastructural method after live imaging could then finally provide confirmation of bud morphology. Finally, an additional difficulty is that the cargo of these NEB pathways may only transiently exist in the cytoplasm before being degraded, further complicating the experimental design.

Unanswered NEB questions:

- Is NEB truly a transport of molecules across the nuclear envelope? How can this be experimentally verified?
- Do all NEB events transport cargo via the same mechanism, or are there different transport pathways that have overlapping nuclear envelope dynamics?
- Are multiple cargoes transported in the same NEB pathway or are they selective for certain substrates?
- Is the transport uni- or bi-directional?
- What is the destination of cargo that utilizes these various NEB pathways?

All NEB events have certain features in common, such as deformation of one or both nuclear membranes and the fact that they contain cargo. Yet, there are ultrastructural differences between them. RNA granules found in *D. melanogaster* protrude into the cell nucleus and contain multiple, electron-dense granules. Those individual granules are surrounded by a single membrane, possibly INM [28]. NEB events in the yeast *S. cerevisiae*, on the other hand, point outward of the nucleus, protruding into the cytoplasm. The cargo is not always membrane-encapsulated and has different electron densities. Sometimes the appearance of cargo on micrographs resembles that of the nucleoplasm, and other times that of the cytoplasm [27]. In the same organism, when the transport of cargo is mediated by Atg39 under autophagy-inducing conditions, multiple vesicles consisting of INM and nuclear material accumulate. Together, they form a network covered in ONM, adjacent to the nuclear envelope. Lastly, nuclear envelope herniations that transport defective NPC units, such as in the NPC mutant *nup116*∆, have yet another morphology [121]. Those buddings have a partial NPC at their base, connecting it to the nucleoplasm, in contrast to NEB formed during cellular stress, which are often detached from the INM. These nuclear envelope herniations also appear rather elongated, compared to those observed containing other cargoes such as aggregated proteins. Additionally, immuno-EM on healthy cells shows only partial labeling of Nups on NEB events [27]. This indicates that not all NEB events are formed around a partially assembled and/or defective NPC.

The fact that there is variety in the appearance of NEB events poses the question of whether all observations fall under the same cellular pathway. The membrane surrounding the cargo could be less visible due to technical reasons. For example, the position where the NEB event was sectioned could obscure a membrane. However, the fact that NEB events without visible membrane have been observed using electron tomography [27] increases the probability that membrane-less cargo can accumulate between the nuclear membranes.

It is also unclear whether NEB events are transporting a single cargo with its own NEB pathway or a mixture of different cargoes in the same event. It should be noted that ubiquitin, although observed both in *S. cerevisiae* and human NEB events [27,127–129], does not localize to the NEB events in *D. melanogaster* muscle nuclei (Speese – unpublished observations), which is consistent with the idea that different types of cargo can be ferried across the nuclear envelope by NEB.

The directionality of NEB transport is hard to establish in static imaging studies. In Speese *et al.* [28], it was hypothesized that NEB was proceeding out of the nucleus due to the nature of the RNA content, but this remains unclear in electron microscopic studies when only a snapshot in time can be visualized. A vesicle about to fuse with the nuclear envelope and one about to bud off would look very similar. Since fusion of membranes is a faster process than that of forming a bud [28,30,183–185], one would be more likely to observe the latter event. This implies that a large fraction of the visible buds would be in the process of forming and not fusing. NEB events observed within the perinuclear space could in principle be transported either way. Thus, only the appearance of the cargo gives some sort of indication of where the cargo originates from. A small subset of NEB events in *S. cerevisiae* contained ribosomes and cargo with the texture of the cytoplasm [27]. This could indicate that the NEB transport route also allows a way into the nucleus. As a portion of cellular proteasomes is localized within the nucleus, this is an important site for proteasomal removal of damaged proteins. Cytoplasmic proteins can be shuttled to the nucleus for proteolytic breakdown [186–188]. Whether these proteins are potentially transported to the nucleus already as aggregates is unclear, but large protein aggregates would struggle to traverse the NPC due to their size restrictions.

In the case of cargo being transported out of the nucleus, the most elusive step of the NEB pathway is the final budding event across the ONM into the cytoplasm. It is not clear whether the cargo will be released inside a vesicle or membrane-free into the cytoplasm via a fusion event, like in the viral egress of herpes viral nucleocapsids. Various flavors of NEB pathways may solve this last step differently, and it is thus important to remain open-minded as to what the final destination of the cargo moving through the various NEB pathways may be. The fate of the cargo once in the cytoplasm also remains elusive, but it might be removed via autophagy or fed into proteasomes for degradation. NEB events formed upon nutritional stress to aid the turnover of INM are engulfed into autophagosomes and shuttled to the vacuole for autophagic degradation [30,107]. The proteome of the nuclear envelope is safeguarded by dedicated degradation machineries, including the INM-associated degradation (INMAD) as well its equivalent at the ONM and the continuous ER membrane, the ER-associated degradation (ERAD). These machineries retrotranslocate misfolded or orphan proteins for subsequent removal by the proteasome [189–191]. Here, NEB seems to contribute to supporting the INM integrity by sequestering its damaged parts for turnover via autophagy. How the NEB pathway is embedded into a broad spectrum of protein quality control systems that maintain the integrity and function of the nucleus and the nuclear envelope remains to be fully understood. However, NEB pathways seem to represent an important mode of nuclear protein quality control that safeguard proteostasis when one subsystem fails for instance, upon insufficient function of the proteasome [27].

With links to several diseases proposed, such as laminopathies, cancer, and neurodegenerative disorders, the acceptance of NEB pathways as an alternative route across the nuclear envelope will open up a whole range of new important questions concerning potential cargoes, molecular components, mechanisms, and cellular destinations.
